# Prediction of the Complication Risk in Drug-Resistant Tuberculosis After Surgery: Development and Assessment of a Novel Nomogram

**DOI:** 10.3389/fsurg.2021.689742

**Published:** 2021-08-09

**Authors:** Liwei Wu, Xiyong Dai, Haijiang Wang, Chaolin Huang, Fan Xia, Yanzheng Song, Lin Wang

**Affiliations:** ^1^Department of Thoracic Surgery, Shanghai Public Health Clinical Center, Fudan University, Shanghai, China; ^2^Department of Surgery, Wuhan Pulmonary Hospital, Wuhan, China; ^3^Department of Thoracic Surgery, The Third People's Hospital of Shenzhen, Shenzhen, China; ^4^Department of Thoracic Surgery, Wuhan Jinyintan Hospital, Wuhan, China; ^5^Department of Pulmonary Disease, 905Th Hospital of PLA Navy, Shanghai, China; ^6^TB Center, Shanghai Emerging and Re-emerging Infectious Disease Institute, Fudan University, Shanghai, China

**Keywords:** tuberculosis, nomogram, surgery, LTBS classification system, drug resistance tuberculosis

## Abstract

**Background:** Surgery is increasingly accepted as an adjunctive approach to treat multidrug-resistant tuberculosis (MDR-TB) or extensively drug-resistant tuberculosis (XDR-TB). However, a model that includes all factors to predict the risk of postoperative complications is lacking.

**Methods:** We developed a prediction model based on 138 patients who had undergone surgery as treatment for drug-resistant tuberculosis (DR-TB) after 24 months. Clinical features on the lesion type (L), treatment history (T), physiologic status of the body (B), and surgical approach (S) were evaluated. Multivariable logistic regression analysis was conducted by clinical features selected in the least absolute shrinkage and selection operator (LASSO) to build a nomogram. The discrimination, calibration, and clinical usefulness of the nomogram were assessed using the C-Index, calibration plots, and decision curves. Internal validation was assessed using bootstrapping.

**Results:** The nomogram contained the features L, B, T, cavitary, recurrent chest infection (RCI) and MDR-TB/XDR-TB. The model displayed good discrimination with a C-Index of 0.879 (95% CI: 0.799–0.967). A high C-Index of 0.824 was achieved in the interval validation. Decision-curve analysis showed that the nomogram was clinically useful if intervention was decided at the non-adherence possibility threshold of 4%.

**Conclusion:** Our novel nomogram could be used conveniently to predict postoperative complication risk in DR-TB patients.

## Key points

- We developed a new nomogram to predict the risk of postoperative complications.
- This nomogram was accurate in predicting the risk of postoperative complications.
- This nomogram contains six factors that can be obtained readily for prediction.
- This nomogram can reduce the risk of surgical complications in patients with tuberculosis.

## Introduction

Tuberculosis remains a major public-health problem. The emergence of drug-resistant tuberculosis (DR-TB) makes the control of tuberculosis more difficult. Patients with multidrug-resistant tuberculosis (MDR-TB) or extensively drug-resistant tuberculosis (XDR-TB) have a high mortality rate and consume considerable medical resources (1). Surgery was considered effective treatment of tuberculosis before the emergence of efficacious anti-tuberculosis drugs (2). With the emergence of anti-tuberculosis drugs, surgery became a less prominent form of treatment. In recent years, with the emergence of DR-TB, surgery has once again become adjunctive therapy for tuberculosis.

According to guidelines set by the World Health Organization, ≥2 months of anti-tuberculosis treatment is required before surgical treatment (1, 3). However, those guidelines also reviewed relevant studies, and found a lack of basic evidence for the risk of complications after surgical treatment of DR-TB (4, 5). Therefore, evidence of the risk of postoperative complications of DR-TB is needed. However, postoperative complications are affected by multiple preoperative factors: treatment-related factors (duration of preoperative anti-tuberculosis treatment, medication regimen, the number and type of drugs that *Mycobacterium tuberculosis* is resistant to), physiology-related factors [body mass index, diabetes mellitus (DM)], treatment and patient-related factors (age, sex, distribution of lesions, clinical symptoms) (5, 6).

An accurate prediction model that can include various factors is necessary. Current guidelines focus on several factors, but do not include all factors, to predict the risk of postoperative complications. A model that includes all factors to predict the risk of complications after surgery for tuberculosis disease is lacking.

We wished to develop a valid (but simple) prediction tool for DR-TB patients to assess the risk of complications using only the characteristics readily available upon therapy initiation.

## Materials and Methods

### Ethical Approval of the Study Protocol

The study protocol was approved by the Ethics Committee of Shanghai Public Health Clinical Center (Shanghai, China). Written informed consent was obtained before patient selection.

### Clinical Data

All 138 MDR/XDR-TB patients who had received surgical intervention as treatment were evaluated retrospectively. Treatment was provided between October 2010 and August 2016. All patients had been diagnosed in regional hospitals to have resistance to rifampicin and/or isoniazid, and had been treated before being referred to one of six hospitals in China for further management: Shanghai Public Health Clinical Center, Third People's Hospital of Shenzhen, Wuhan Pulmonary Hospital, 905^th^ Hospital of PLA Navy, and Wuhan Jinyintan Hospital.

### Inclusion and Exclusion Criteria

The inclusion criteria were as follows: persistent positive (or repeat positive) findings of *M. tuberculosis* in sputum; cavitation and destruction of the lung; patients who had undergone anti-tuberculosis chemotherapy had been followed up for 3 months; radiography and computed tomography of the chest showed lesions.

The exclusion criteria were: the duration of anti-tuberculosis chemotherapy was <2 months; patients with mental illness who could not understand the study requirements; cerebrovascular disease, hypertension, DM, and other surgical contraindications; poor compliance.

### Establishment of the “LTB-S” Classification System

Based on our previous research (2), we defined LTB-S as a classification system for surgical therapy of tuberculosis. The “L” denotes tuberculosis lesions, “T” refers to treatment history, “B” denotes the physiological status of patients, and “S” denotes the surgical approach (Table 1).

**Table 1:** Description of LTBS Classification system.

L	Lesion(s): 3 types defined by radiology imaging	
	L1	Stable, localized, unilateral/bilateral lesions.
	L2	Diffused, progressive unilateral lesion(s), with or without stable lesions on the other lobe.
	L3	Diffused, progressive bilateral lesions; or active/progressive extra pulmonary tuberculosis was found.
T	Treatment history: 3 types defined by clinical record	
	T1	A standard chemotherapy cycle (1) for MDR/XDR-TB was unfinished but more than 2 months before surgery.
	T2	More than one standard chemotherapy cycle for MDR/XDR-TB was completed before surgery.
	T3	A standard chemotherapy regimen for MDR/XDR-TB was given less than 2 months, or no standard chemotherapy regimen was given before surgery.
B	Body: 3 classes defined through specific physiological examination before surgery, assessing general condition, potential complications, along with symptoms of tuberculosis intoxication.	
	B1	Good general condition, no physiological complication, no obvious symptoms of tuberculosis poisoning.
	B2	Permissible general condition, or with mild underlying diseases such as diabetes, high blood pressure, coronary artery disease, chronic liver diseases and so on which can be conventionally managed and controlled, or with mild symptoms of tuberculosis poisoning.
	B3	Poor general condition, or with severe physiological complications, or with evident symptoms of tuberculosis intoxication.
S	Surgery: 2 types defined by surgery approach.	
	S0	Radical surgery: no tuberculosis foci in other locations are left after resection of localized and stable primary lesions.
	S1	Palliative operation: lesions are present at multiple sites. Stable lesions remain after resection.

### Definition of Postoperative Complications

The postoperative complications were bronchopleural fistulae, bleeding, contralateral spread, prolonged air leak, infection, and formation of a residual cavity. The duration of follow-up to observe postoperative complications was 30 days.

### Statistical Analyses

Data (demographic, disease, treatment characteristics) are expressed as counts (%). Statistical analyses were undertaken using R 3.6.2; (www.R-project.org).

The least absolute shrinkage and selection operator (LASSO) method was used to select the optimal predictive features from patients with DR-TB. This method is suitable for high-dimensional data reduction (7, 8). Features with non-zero coefficients in the LASSO regression model were selected (9). Then, the features which were applied to develop a prediction model were selected manually from the selection features of LASSO. Based on the selected features, a prediction model was established by multiple logistic regression analysis. The features were considered as odds ratio (OR) having a 95% confidence interval (CI) and *P*-value. The level of statistical significance was two-sided. The features associated with disease characteristics and treatment characteristics were included (10). A prediction model was established by applying all the potential prediction features and using a patient cohort (11, 12).

Curves were plotted to assess the calibration of the nomogram (13). Harrell's Concordance Index was employed to quantify the discrimination performance of the nomogram. The nomogram was subjected to bootstrapping validation (1,000 bootstrap resamples) to calculate a relatively corrected Concordance Index (14). Analysis of decision curves was conducted to determine the clinical usefulness of the nomogram by quantifying the net benefits at different threshold probabilities in the DR-TB cohort (15). The net benefit was calculated by subtracting the proportion of all patients who were false-positive from the proportion of patients who were true-positive, and by weighing the relative harm of forgoing interventions compared with the negative consequences of an unnecessary intervention (16).

## Results

### Characteristics of Patients

A total of 138 patients (99 males and 39 females) visiting six hospitals from October 2010 to January 2015 underwent a surgical procedure. The cohort consisted of 116 patients with MDR-TB and 22 patients with XDR-TB. Recurrent chest infection (RCI) was documented in 38 patients, and cavitary pulmonary tuberculosis in 53 cases. Lobectomy was undertaken in 104 patients (75.4%), pneumonectomy in 14 individuals (10.1%) and wedge resection in 20 cases (14.5%). A total of 108 cases did not suffer postoperative complications. Thirty cases suffered complications: bronchopleural fistula (*n* = 4), wound infection (*n* = 7), space problem (*n* = 11), prolonged air leak (*n* = 7), and intrathoracic bleeding necessitating re-exploration (*n* = 2). Of these, five patients experienced two complications. Underlying diseases before surgery were DM (*n* = 9), cardiac insufficiency (*n* = 5), chronic liver diseases (*n* = 3), chronic obstructive pulmonary disease or other lung diseases (*n* = 2), hypertension (*n* = 2), coronary heart disease (*n* = 1), and laryngeal phthisis (*n* = 1).

Patients were divided into two groups according to whether they had postoperative complications. All data of patients (demographic, disease, and treatment features) in the two groups are given in Table 2.

**Table 2:** Demographic and clinical characteristics of complication and non-complication groups.

**Characteristics**	***n*** **(%)**		
	**No complication**	**Complication**	**Total**
	**(*n* = 108)**	**(*n* = 30)**	**(*n* = 138)**
Sex			
Female	26 (24.1)	13 (43.3)	39 (28.3)
Male	82 (75.9)	17 (56.7)	99 (71.7)
Age			
<55	91 (84.3)	27 (90.0)	118 (85.5)
≥55	17 (15.7)	3 (10.0)	20 (14.5)
L			
L1	74 (68.5)	11 (36.7)	85 (61.6)
L2	32 (29.6)	13 (43.3)	45 (32.6)
L3	2 (1.9)	6 (20.0)	8 (5.8)
T			
T1	89 (82.4)	19 (63.3)	108 (78.3)
T2	19 (17.6)	7 (23.3)	26 (18.8)
T3	0 (0.0)	4 (13.3)	4 (2.9)
B			
B1	73 (67.6)	13 (43.3)	86 (62.3)
B2	35 (32.4)	15 (50.0)	50 (36.2)
B3	0 (0.0)	2 (6.7)	2 (1.4)
S			
S0	71 (65.7)	11 (36.7)	82 (59.4)
S1	37 (34.3)	19 (63.3)	56 (40.6)
Side			
Unilateral	78 (72.2)	12 (40.0)	90 (65.2)
Bilateral	30 (27.8)	18 (60.0)	48 (34.8)
Cavitary			
No	73 (67.6)	12 (40.0)	85 (61.6)
Yes	35 (32.4)	18 (60.0)	53 (38.4)
Hemoptysis			
No	83 (76.9)	17 (56.7)	100 (72.5)
Yes	25 (23.1)	13 (43.3)	38 (27.5)
RCI			
No	88 (81.5)	12 (40.0)	100 (72.5)
Yes	20 (18.5)	18 (60.0)	38 (27.5)
MDR/XDR			
MDR	97 (89.8)	19 (63.3)	116 (84.1)
XDR	11 (10.2)	11 (36.7)	22 (15.9)
Sputum Culture			
Negative	62 (57.4)	11 (36.7)	73 (52.9)
Positive	46 (42.6)	19 (63.3)	65 (47.1)

*L, Lesion; T, Treatment history; B, Body condition; RCI, Recurrent chest infection; MDR/XDR, Multi drug resistant/extensively drug resistant*.

### Feature Selection

**Figure 1:**
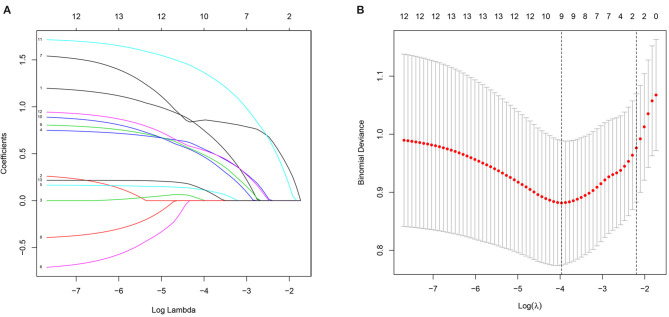
Demographic and clinical feature selection using the LASSO binary logistic regression model. **(A)** LASSO coefficient profiles of the 12 features. A coefficient profile plot was produced against the log (lambda) sequence. Vertical line was drawn at the value selected using fivefold cross-validation, where optimal lambda resulted in nine features with non-zero coefficients. **(B)** Optimal parameter (lambda) selection in the LASSO model used fivefold cross-validation via minimum criteria. The partial likelihood deviance (binomial deviance) curve was plotted vs. log (lambda). Dotted vertical lines were drawn at the optimal values by using the minimum criteria and the 1 SE of the minimum criteria (the 1-SE criteria).

Based on demographic, disease, and treatment features, 13 features were reduced to nine potential features and were given non-zero coefficients in the LASSO regression model (Figures 1A,B). Combining the results of LASSO analysis and multiple logistic analysis, the following six features were selected: L, B, T, cavitary, RCI, and MDR-TB/XDR-TB.

### Development of an “Individualized” Prediction Model

**Figure 2:**
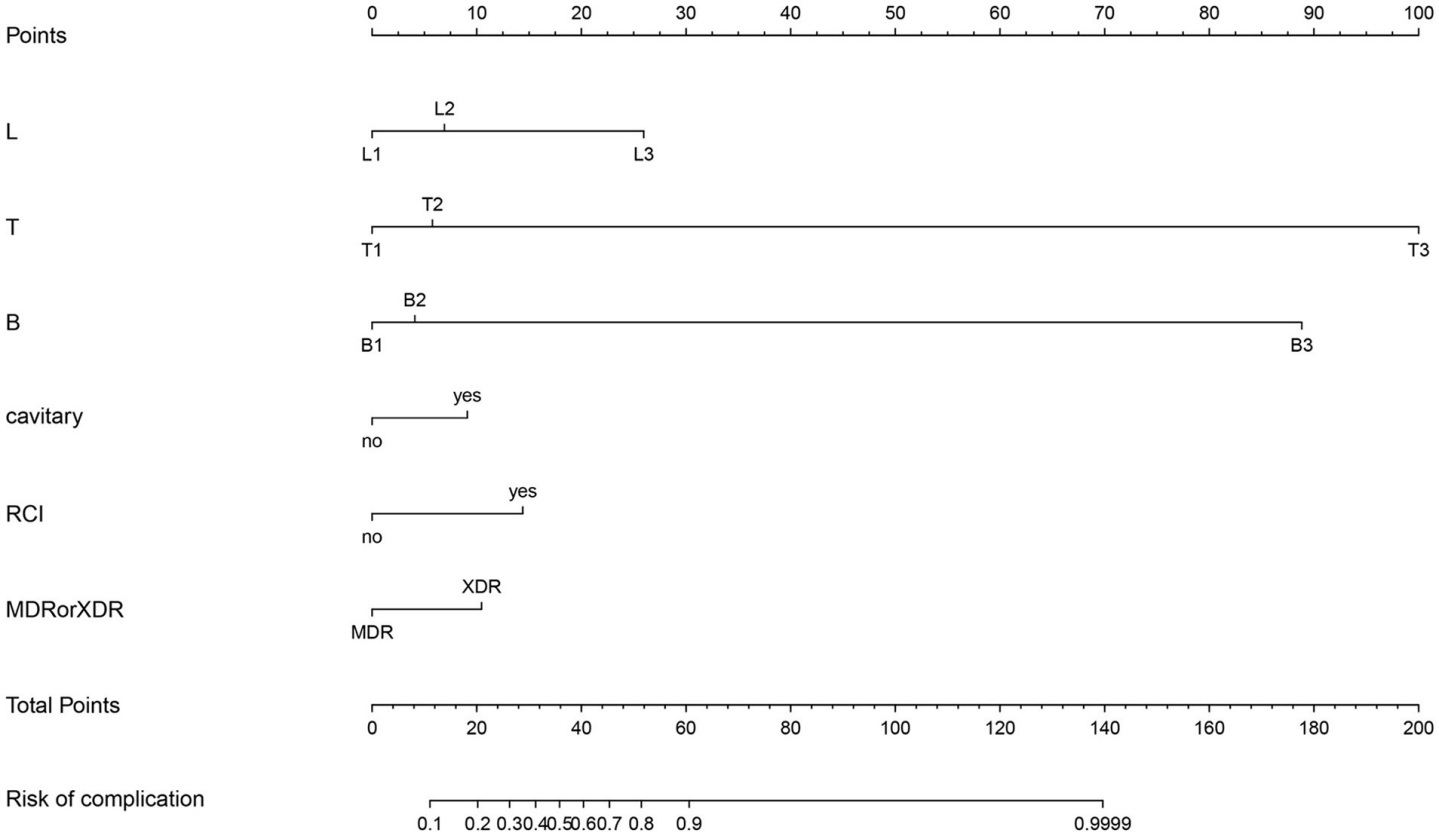
Developed postoperative complication nomogram. L, Lesion; T, Treatment history; B, Body condition; RCI, Recurrent chest infection; MDR/XDR, Multi drug resistant/extensively drug resistant.

The results of the multiple logistic regression analysis among the independent predictors (L, B, T, cavitary, RCI, and MDR-TB/XDR-TB) are given in Table 3. The model that incorporated the independent predictors stated above was developed and is presented as the nomogram in Figure 2.

**Table 3:** Prediction factors for postoperative complication in DR-TBs.

**Intercept and variable**	**Prediction model**		
	**β**	**Odds ratio (95% CI)**	***P*-value**
Intercept	−9.084	0.0001 (0.000003–0.0021)	<0.001
L	0.871	2.387 (1.078–5.489)	0.034
T	1.146	3.141 (1.289–8.229)	0.013
B	0.575	1.777 (0.687–4.715)	0.236
Cavitary	0.813	2.255 (0.801–6.451)	0.122
RCI	1.401	4.057 (1.413–12.031)	0.009
MDR\XDR	0.819	2.268 (0.654–7.601)	0.186

### Performance of the Nomogram for Postoperative Complication Risk in the Cohort

**Figure 3:**
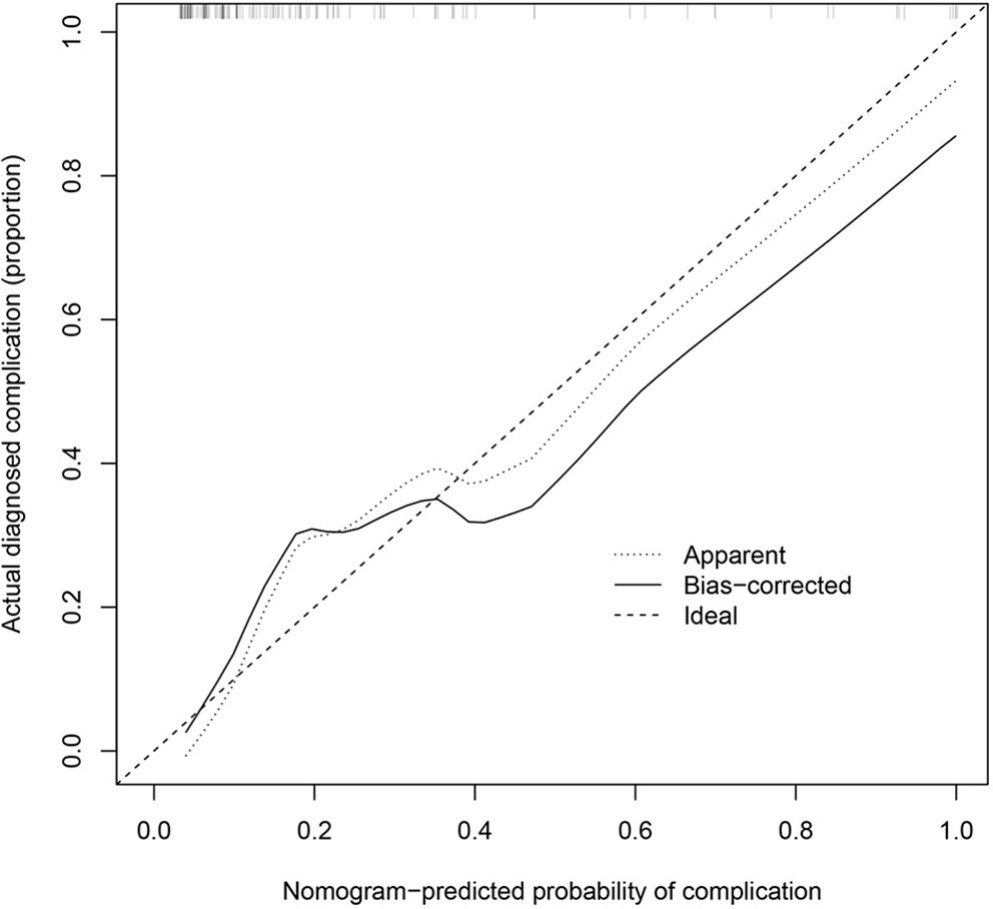
Calibration curves of the nomogram prediction in the cohort. The x-axis represents the predicted postoperative complication risk. The y-axis represents the actual happened complication. The diagonal dotted line represents a perfect prediction by an ideal model. The solid line represents the performance of the nomogram, of which a closer fit to the diagonal dotted line represents a better prediction.

The calibration curve of the nomogram for the prediction of postoperative complication risk in DR-TB patients demonstrated a good performance (Figure 3). The Concordance Index for the prediction nomogram was 0.879 (95% CI: 0.799–0.967) for the cohort, and was confirmed to be 0.824 through bootstrapping validation, which suggested the good discrimination of this nomogram.

### Clinical Use

**Figure 4:**
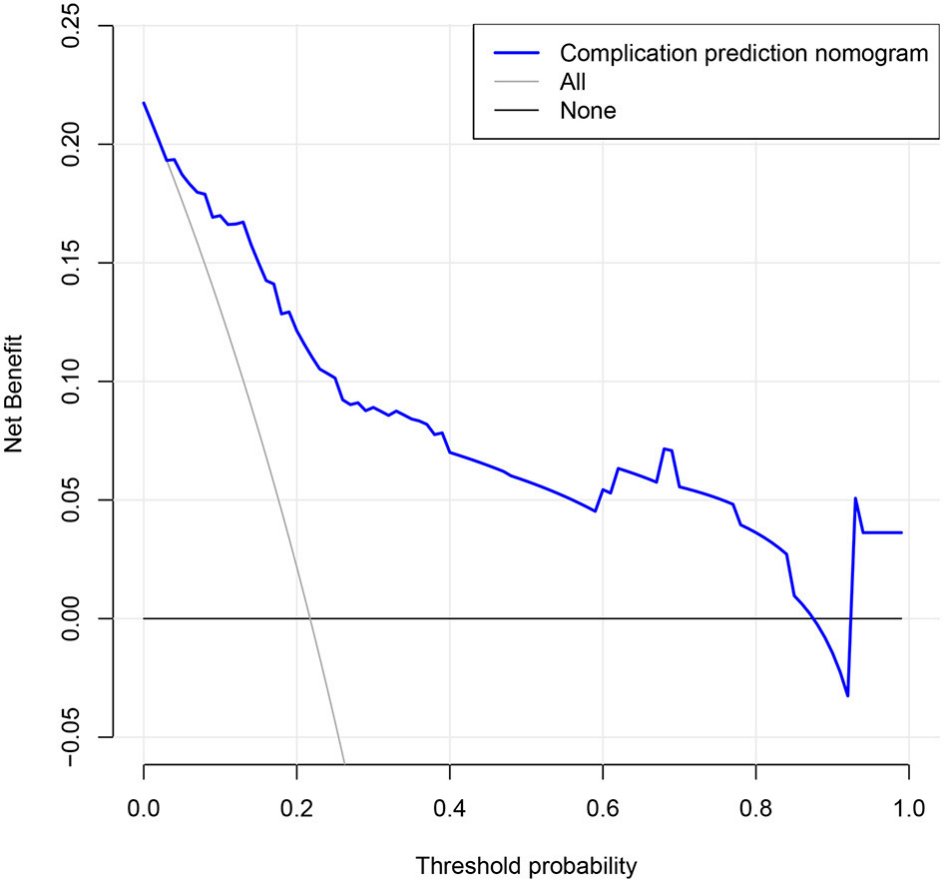
Decision curve analysis for the nomogram. The y-axis measures the net benefit. The blue line represents the postoperative complication risk nomogram. The thin solid line represents the assumption that all patients get complication after surgery. Thin thick solid line represents the assumption that no patients get complication after surgery. The decision curve showed that if the threshold probability of a patient and a doctor is 4 and 86%, respectively, using this nomogram in the current study to predict postoperative complication risk adds more benefit than the intervention-all-patients scheme or the intervention-none scheme.

The analysis for the decision curve for the nomogram is presented in Figure 4. The decision curve showed that if the threshold probability of a patient and a physician was 4 and 86%, respectively, use of our nomogram to predict the risk of postoperative complications added more benefit than the scheme. Within this range, net benefit was comparable with several overlaps on the basis of our nomogram.

## Discussion

Nomograms are employed widely in medicine and oncology (17). Thanks to a user-friendly interface and good prediction performance, a nomogram can help clinicians to make important decisions. We, for the first time, used a nomogram to predict the risk of postoperative complications in patients with DR-TB.

We developed and validated a new predictive model that used six readily available variables to predict the risk of postoperative complications in patients with DR-TB. Combining demographic, disease, and treatment risk factors into a single nomogram helps to “personalize” the prediction in DR-TB patients. We provided a relatively accurate tool for predicting the risk of postoperative complications for patients with DR-TB. The internal verification in the cohort demonstrated good discrimination and calibration ability. In particular, the high Concordance Index in the interval verification demonstrated that the nomogram could be used widely and elicit accurate results in a large cohort (18, 19).

In the present study, 21.7% of patients had postoperative complications. In the analysis of risk factors, L, B, T, cavity, RCI, and MDR-TB/XDR-TB were associated with the risk of postoperative complications. Patients with L1, B1, T1, no cavity, no RCI, and MDR-TB were less likely to have postoperative complications (Figure 3). These may be the key individual factors of postoperative complications in patients. Similar to our previous studies, L1 patients had the lowest risk of postoperative complications. That is, lesions suitable for resection should be localized, whether on the unilateral side or bilateral sides of the lungs (classified as “L1”). This conclusion is consistent with the conclusion in studies that reviewed evidence of localized diseases as a standard for resection, which suggested that patients with localized lesions before and after surgery can gain more benefits (20, 21).

T1 patients carried the lowest risk of postoperative complications. We showed that, as long as standardized anti-tuberculosis treatment was >2 months before the surgical procedure, the latter was safe and efficacious. The World Health Organization recommends ≥2 months of anti-tuberculosis treatment before surgery. However, efficacious anti-tuberculosis treatment preoperatively can inhibit the number and activity of *M. tuberculosis* and reduce the risk of complications (3). However, for a small number of patients, excessive prolongation of the duration of preoperative chemotherapy may not be helpful (22). Even if most patients have received adequate preoperative chemotherapy, the lesion can worsen (classified as “L3”) or the general condition can deteriorate (classified as “B2” or “B3”). Therefore, for these patients, preoperative chemotherapy is not efficacious. However, the results of our study suggest that surgical treatment can be considered after ≥2 months of anti-tuberculosis treatment. This strategy can not only ensure the lowest probability of postoperative complications, it can also reduce the physical/psychological injury and economic burden caused by the side-effects of anti-tuberculosis drugs.

The physical status of the patient must also be considered. Although the physical condition cannot be quantified in our system, it can reflect the general physical status of the patient before surgery. In our study, the risk of complications was lowest when patients had good physical condition, with no physiological complications or obvious symptoms of tuberculosis poisoning. Similar to observations in other studies, patients in good health are more tolerant of surgery (22, 23). Patients without RCI or lung cavities also carry the lowest postoperative risk which, to some extent, is analogous to category B1. Patients with MDR-TB had the lowest postoperative risk because patients with MDR-TB are, in general, in good physical condition and have few pathologic changes. However, there was no significant difference in the score for MDR-TB cases and XDR-TB patients in our nomogram. This observation may indicate no significant difference in the risk of postoperative complications between MDR-TB cases and XDR-TB patients if other conditions are identical.

DR-TB is treated mainly with drugs. However, treatment of DR-TB is usually long-term, which can result in many adverse reactions to drugs and a heavy economic burden. If patients cannot tolerate standardized anti-tuberculosis treatment, then treatment failure is likely. If surgical treatment is adopted, it can not only shorten the course of treatment, but also reduce the cost of treatment. In this context, the control of postoperative complications is particularly important. Although DR-TB may have been cured, most patients do not accept that their physical condition has been worsened by postoperative complications. We developed an effective risk-prediction tool that can help clinicians assess the risk of postoperative complications accurately. Through use of six indicators, we can make a quantitative prediction of the risk of postoperative complications as a whole, rather than relying on a single index. The prediction model is unified and integrated, and all related factors are interrelated and interact with each other. Therefore, accurate prediction of the risk of postoperative complications can help clinicians to choose surgery at an appropriate time and avoid postoperative complications. Use of our nomogram to select patients suitable for surgery accurately makes surgical treatment of DR-TB more personalized.

The present study had two main limitations. First, as a limitation of retrospective studies, all data were from patients, so many indicators are difficult to quantify accurately. Second, although the robustness of our nomogram was tested extensively by internal verification and Bootstrap testing, it was not verified externally: external evaluation in wider populations of DR-TB is needed.

## Conclusions

We developed a novel nomogram with relatively good accuracy to help clinicians assess the risk of postoperative complication in DR-TB patients when initiating a surgical procedure. With an estimate of individual risk, clinicians and patients can take appropriate decisions on medical interventions. This nomogram requires external validation, and further research is needed to determine if individual interventions based on this nomogram will reduce the risk of postoperative complications.

## Data Availability Statement

The raw data supporting the conclusions of this article will be made available by the authors, without undue reservation.

## Ethics Statement

The studies involving human participants were reviewed and approved by Ethics Committee of Shanghai Public Health Clinical Center. The patients/participants provided their written informed consent to participate in this study.

## Author Contributions

YS and LWa: Conception and design. LWa, XD, HW, CH, FX, and YS: Acquisition of clinical data. LWu and LWa: Data analysis. LWu, LWa, XD, HW, CH, FX, and YS: Preparation and review of the manuscript. All authors contributed to the article and approved the submitted version.

## Conflict of Interest

The authors declare that the research was conducted in the absence of any commercial or financial relationships that could be construed as a potential conflict of interest.

## Publisher's Note

All claims expressed in this article are solely those of the authors and do not necessarily represent those of their affiliated organizations, or those of the publisher, the editors and the reviewers. Any product that may be evaluated in this article, or claim that may be made by its manufacturer, is not guaranteed or endorsed by the publisher.

## Abbreviations

LTB-S, classification system for resection of pulmonary tuberculosis based on L (lesion), T (treatment history): B (physiologic status of the body) and S (surgery)
RCI: recurrent chest infection
MDR-TB: multi-drug resistant tuberculosis
XDR-TB: extensively drug-resistant tuberculosis
DR-TB: drug-resistant tuberculosis
LASSO: least absolute shrinkage and selection operator
OR: odds ratio
CI: confidence interval
RCI: recurrent chest infection.
